# EXAMINING THE ROLE OF SOUTHERN CONTEXT FOR DEMENTIA, DISABILITY, AND MORTALITY AMONG OLDER US BLACKS AND WHITES

**DOI:** 10.1093/geroni/igae098.1495

**Published:** 2024-12-31

**Authors:** Jaein Kim, Hyungmin Cha, Mateo Farina, Mark D Hayward

**Affiliations:** Population Research Center, The University of Texas Austin, Austin, Texas, United States; University of Southern California, Los Angeles, California, United States; University of Texas at Austin, Austin, Texas, United States; The University of Texas Austin, Austin, Texas, United States

## Abstract

Racial inequalities in older adult health are well-documented. Compared to Whites, Blacks have greater risks of disability, dementia, multimorbidity and mortality. These differences in age-related health are paralleled by race differences in context. In particular, approximately 80% of older Blacks were born in Jim Crow South no matter where they currently live compared to 30% percent of older Whites. In the United States, the U.S. South has historically structured the life course experiences of Black and White older adults differently through legalized racial segregation. As a result, the role of association of Southern context with later life health risks is expected to differ substantially across the groups. We focus on three critical dimensions of population health to better understand the role of context for race difference in health – cognitive and physical functioning as well as mortality. Drawing on the Health and Retirement study from 2000-2016, we estimate a series of hazard models for each of the health outcomes separately for each race group. This approach promises new insights into the role of context for within-group variation in the health outcomes as well as for potential between-group differences for each specific outcome. Whereas for Whites Southern birth regardless of residence was associated with increased risk across all three conditions, for Black older adults we found more heterogeneity. Those who were born and lived in the South had the highest risk of dementia. Southern residence was associated with an increased risk of mortality, but its effect was ambiguous for risk of disability.

